# Plasminogen Activating Inhibitor-1 Might Predict the Efficacy of Anti-PD1 Antibody in Advanced Melanoma Patients

**DOI:** 10.3389/fonc.2021.798385

**Published:** 2021-11-29

**Authors:** Kentaro Ohuchi, Yumi Kambayashi, Takanori Hidaka, Taku Fujimura

**Affiliations:** Department of Dermatology, Tohoku University Graduate School of Medicine, Sendai, Japan

**Keywords:** melanoma, PAI-1, TAMs, Anti-PD1 Abs, efficacy

## Abstract

Plasminogen activating inhibitor-1 (PAI-1) plays crucial roles in the development of various cancers, including melanomas. Indeed, various pro-tumorigenic functions of PAI-1 in cancer progression and metastasis have been widely reported. Among them, PAI-1 is also reported as a key regulator of PD-L1 expression on melanoma cells through endocytosis, leading to abrogating the efficacy of anti-PD1 antibodies (Abs). These findings suggested that PAI-1 expression might predict the efficacy of anti-PD1 Abs. In this report, the expression and production of PAI-1 in melanoma patients were evaluated, and the immunomodulatory effects of PAI-1 on tumor-associated macrophages were investigated *in vitro*. Immunohistochemical staining of PAI-1 showed that PAI-1 expression on melanoma cells was significantly decreased in responders compared to non-responders. Moreover, baseline serum levels of PAI-1 were significantly decreased in responders compared to non-responders. Notably, PAI-1 decreased the production of various chemokines from monocyte-derived M2 macrophages *in vitro*, suggesting that PAI-1 might decrease tumor-infiltrating lymphocytes to hamper the anti-tumor effects of anti-PD1 Abs. These results suggest that baseline serum levels of PAI-1 may be useful as a biomarker for identifying patients with advanced cutaneous melanoma most likely to benefit from anti-melanoma immunotherapy.

## Introduction

Plasminogen activating inhibitor-1 (PAI-1) is a serine protease that plays crucial roles in the development of various cancers, including melanomas (1–3). PAI-1 inhibits urokinase-type plasminogen activator and tissue type plasminogen activator, leading to attenuation of plasminogen activation and the development of thrombosis formation at tumor sites. PAI-1 increases the expression of focal adhesion kinase on tumor-associated macrophages (TAMs) to facilitate the migration of macrophages into tumor sites in a melanoma model (1). More recently, PAI-1 was found to facilitate PD-L1 endocytosis of melanoma cells to abrogate the efficacy of anti-PD-L1 antibodies (Abs) in mouse melanoma models (2). In addition, PAI-1 was found to induce resistance to chemotherapy in mouse B16F10 melanoma (3). These reports suggested that PAI-1 could play a significant role in maintaining the immunosuppressive microenvironment in melanoma through TAMs. In this study, the expression and production of PAI-1 were evaluated in melanoma patients, and the immunomodulatory effects of PAI-1 on TAMs were investigated *in vitro*.

## Patients and Methods

### Ethics Statement for Human Experiments

The protocol for this human study was approved by the ethics committee of Tohoku University Graduate School of Medicine, Sendai, Japan (permit no. 2020-1-759). All methods were performed in accordance with the relevant guidelines and regulations. All patients provided written, informed consent prior to enrolment in the study.

### Patients

Patients were eligible if they had unresectable stage III melanoma or if they had stage IV melanoma with accessible cutaneous, subcutaneous, and/or nodal lesions (staging was performed according to the AJCC Staging Manual, 8th edition, 2018). All patients received 2 mg/kg of nivolumab every three weeks, 3 mg/kg of nivolumab every two weeks, 240 mg of nivolumab every two weeks, 480 mg of nivolumab every four weeks, or 200 mg of pembrolizumab every three weeks. Serum was obtained from patients before the administration of anti-PD1 Abs. The response to anti-PD1 Abs was assessed according to the Response Evaluation Criteria In Solid Tumors.

### Tissue Samples and Immunohistochemical Staining

Polyclonal rabbit Abs for human PAI-1 (Abcam, Tokyo, Japan) were used for immunohistochemical (IHC) staining. Archival formalin-fixed paraffin-embedded skin specimens were collected at the initial visit from 30 advanced melanoma patients who were treated with anti-PD1 Abs in the Department of Dermatology at Tohoku University Graduate School of Medicine. The patients’ characteristics are summarized in **Table 1**.

**Table 1:** Characteristics and PAI-1 expression levels of 31 patients with advanced melanomas.

	Age (y)	Sex	Location	Clark’s classification	Bastian’s classification	Efficacy	PAI-1
1	81-90	F	cheek	NM	CSD	PR	2+
2	71-80	F	toe	ALM	Acral	CR	2+
3	71-80	M	shoulder	NM	non-CSD	PD	–
4	71-80	M	heel	ALM	Acral	PD	–
5	81-90	F	toe	ALM	Acral	PR	2+
6	81-90	M	sole	ALM	Acral	PD	–
7	51-60	M	back	NM	non-CSD	PD	1+
8	71-80	M	toe	ALM	Acral	PD	–
9	81-90	F	sole	ALM	Acral	PD	2+
10	71-80	F	femor	SSM	non-CSD	PD	–
11	71-80	F	toe	ALM	Acral	PD	2+
12	81-90	F	back	SSM	non-CSD	PD	–
13	61-70	M	toe	ALM	Acral	PR	2+
14	71-80	M	sole	ALM	Acral	PR	2+
15	91-100	M	sole	ALM	Acral	PD	2+
16	81-90	M	lower leg	NM	non-CSD	SD	2+
17	51-60	F	lip	LMM	CSD	PD	–
18	31-40	M	cervical	NM	CSD	PD	2+
19	31-40	F	lower leg	SSM	non-CSD	PR	1+
20	31-40	M	lower leg	NM	non-CSD	PD	–
21	71-80	F	sole	ALM	Acral	PD	2+
22	61-70	F	head	NM	CSD	CR	2+
23	61-70	M	back	NM	non-CSD	PR	2+
24	71-80	M	ear	NM	CSD	PD	–
25	71-80	F	toe	ALM	Acral	PR	2+
26	41-50	M	head	NM	CSD	PD	1+
27	71-80	F	heel	ALM	Acral	PD	–
28	61-70	M	sole	ALM	Acral	PR	1+
29	61-70	F	back	NM	non-CSD	PR	2+
30	31-40	M	cheek	NM	CSD	PD	–
31	41-50	M	lower leg	NM	non-CSD	PD	–

ALM, acral lentiginous melanoma; NM, nodular melanoma; SSM, superficial spreading melanoma; LMM, lentigo maligna melanoma; CSD, cumulative sun damage.

### Quantitative and Semiquantitative Analyses of Immunohistochemical (IHC) Staining

**Figure 1:**
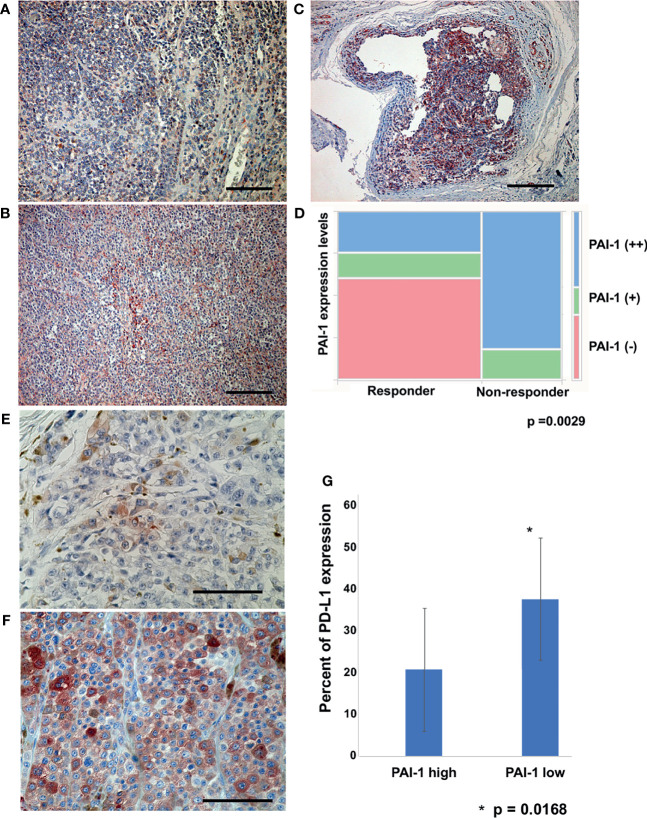
PAI-1-expressing cells in melanoma patients. Representative paraffin-embedded tissue samples from lesional skin of patients with melanoma. Sections were deparaffinized and stained with anti-PAI-1, then developed with liquid permanent red. PAI-1 negative (-) **(A)**, weakly positive (+) **(B)**, and intensely positive (++) **(C)**. Scale bar, 100 µm. Pearson’s correlation coefficient was used to investigate the correlation between therapeutic effect and PAI-1 expression level **(D)**. Sections of melanoma from an anti-PD1 Abs in a high PAI-1 expression patient **(E)** and a low PAI-1 expression patient **(F)** were deparaffinized and stained using anti-PD-L1 antibodies. Sections were developed with liquid permanent red. The percentages of PD-L1+ cells per all melanoma cells were automatically counted by BZ-X800 **(G)**. Scale bar, 100 µm.

For semiquantitative analysis of IHC staining, PAI-1 expression on tumor cells was examined in more than 3 random, representative fields from each section. The expression levels were determined independently by two dermatologists. The expression level of PAI-1 was determined as follows: - (negative: **Figure 1A**), 1+ (weakly positive: **Figure 1B**), and 2+ (intensely positive): **Figure 1C**.

To quantify the IHC staining of each sample, the positive cells were counted using a BZ-X800 (KEYENCE, Tokyo, Japan). The percentage of IHC-positive cells per all tumor-infiltrating cells or melanoma cells was automatically counted.

### Serum PAI-1 Levels

The baseline serum PAI levels were evaluated in 49 patients with advanced melanoma. The serum PAI-1 levels were determined by an enzyme-linked immunosorbent assay (ELISA) according to the manufacturer’s protocol (R&D Systems, Minneapolis, MN). Data from each donor were obtained as the mean of duplicate assays. The protocol for this human study was approved by the ethics committee of Tohoku University Graduate School of Medicine, Sendai, Japan (approval no. 2020-1-759).

### Culturing of M2 Macrophages From Human Peripheral Blood Monocytes

CD14+ monocytes were isolated from the peripheral blood mononuclear cells of healthy donors using MACS beads (CD14 microbeads, Miltenyi Biotec Inc., Sunnyvale, CA) according to the manufacturer’s protocol. The CD14+ monocytes (2 × 10^5^ cells/ml) were cultured in complete medium containing 100 ng/ml of recombinant human macrophage colony-stimulating factor for 5 days, as previously reported (4). On the fifth day, monocyte-derived macrophages were treated with or without PAI-1 (0.1 µg/ml) (5) for 48 hours, and the culture supernatant was harvested. Each chemokine level was determined by an ELISA. This study was approved by the ethics committee of Tohoku University Graduate School of Medicine, Sendai, Japan (2019–1–925).

### Statistical Methods

Pearson’s correlation coefficient was used to investigate the correlation between the therapeutic effect and PAI-1 expression levels. Receiver operating characteristic (ROC) curves were used to calculate cut-off values for serum levels of PAI-1 and areas under the curve (AUCs). Cut-offs were determined using Youden’s index (sensitivity + specificity -1) to determine the maximum index value. ROC curves were established to evaluate serum levels of PAI-1 in patients administered nivolumab. For a single comparison between two groups, the Mann-Whitney U-test was used. All statistical analyses were performed using JMP version 16.1 software (SAS Institute, Tokyo, Japan). The level of significance was set at p<0.05.

## Results

### Demographic Data

Patients’ demographic data of cohort 1 (IHC staining) and cohort 2 (serum PAI-1) are shown in **Tables 1**, **2**, respectively.

**Table 2:** Characteristics and serum levels of PAI-1 in 49 patients with advanced melanomas.

	Age (y)	Sex	Location	Clark’s classification	Bastian’s classification	Efficacy	Serum PAI-1 (pg/ml)
1	81-90	F	cheek	NM	CSD	PD	2.506
2	61-70	M	sole	ALM	Acral	PR	6.285
3	81-90	F	toe	ALM	Acral	PD	4.892
4	71-80	M	lower leg	SSM	non-CSD	SD	3.159
5	81-90	M	head	NM	CSD	PR	2.178
6	51-60	F	upper lip	NM	CSD	PD	2.534
7	31-40	F	lower leg	SSM	non-CSD	SD	6.372
8	71-80	M	groin	NM	non-CSD	PR	2.86
9	71-80	F	vulva	mucosal	mucosal	PD	3.101
10	31-40	M	cheek	LMM	CSD	PD	8.999
11	71-80	F	sole	ALM	Acral	PD	5.034
12	51-60	F	chest	NM	non-CSD	PD	3.781
13	61-70	M	auricle	SSM	CSD	PD	3.288
14	41-50	M	lower leg	SSM	non-CSD	PD	6.657
15	31-40	M	lower leg	NM	non-CSD	PD	2.676
16	71-80	F	toe	ALM	Acral	PR	0.1261
17	59-60	M	femur	NM	non-CSD	PR	2.395
18	41-50	M	sole	ALM	Acral	PD	1.041
19	71-80	F	lower leg	SSM	non-CSD	PR	3.825
20	81-90	M	sole	ALM	Acral	PR	2.486
21	81-90	F	vagina	mucosal	mucosal	PD	2.887
22	61-70	F	back	SSM	non-CSD	PR	2.142
23	41-50	F	vulva	mucosal	mucosal	PR	1.546
24	51-60	F	vagina	mucosal	mucosal	PD	7.166
25	71-80	F	sole	ALM	Acral	PD	3.812
26	71-80	M	sole	ALM	Acral	PR	5.854
27	61-70	F	upper arm	SSM	non-CSD	SD	4.015
28	71-80	M	sole	ALM	Acral	PD	4.402
29	51-60	F	anus	mucosal	mucosal	PD	2.957
30	61-70	M	back	NM	non-CSD	PD	4.647
31	81-90	F	cheek	LMM	CSD	PD	2.8
32	81-90	M	nasal cavity	mucosal	mucosal	PR	2.332
33	41-50	M	cheek	LMM	CSD	PD	3.586
34	71-80	M	sole	ALM	Acral	PD	2.715
35	71-80	M	sole	ALM	Acral	PD	7.636
36	71-80	F	toe	ALM	Acral	PR	3.124
37	61-70	M	penis	NM	non-CSD	PD	4.981
38	81-90	M	sole	ALM	Acral	PD	2.598
39	61-70	M	sole	ALM	Acral	PD	8.59
40	71-80	M	sole	ALM	Acral	PD	5.313
41	41-50	F	sole	ALM	Acral	PD	3.706
42	61-70	F	femur	SSM	non-CSD	SD	5.647
43	41-50	M	shoulder	NM	non-CSD	SD	14.77
44	81-90	F	lower leg	SSM	non-CSD	PR	1.207
45	71-80	M	abdomen	SSM	non-CSD	PD	2.222
46	31-40	F	back	NM	non-CSD	PD	3.938
47	81-90	M	eyelid	NM	CSD	PR	1.539
48	41-50	M	abdomen	SSM	non-CSD	PD	13.97
49	61-70	M	palm	ALM	Acral	PD	2.628

### PAI-1 Expression on Melanoma Correlated With the Efficacies of Anti-PD1 Abs

Since PAI-1 is associated with a poor prognosis in various cancers, and since PAI-1 possesses immunomodulatory effects to polarize TAMs toward proinflammatory and immunosuppressive M2 phenotypes (6, 7), we hypothesized that PAI-1 expression correlates with the efficacies of anti-PD1 Abs for advanced melanoma patients. To test this hypothesis, IHC staining for PAI-1 and semiquantitative analysis of IHC staining of PAI-1 were used. The expression levels of PAI-1 and the best response to anti-PD1 Abs in each patient in cohort 1 are presented in **Table 1**. Pearson’s correlation coefficient showed a significant correlation between the PAI-1 expression level on tumor cells and the best response to anti-PD1 Abs (p=0.0029) (**Figure 1**).

### PD-L1 Expression on Melanoma Correlated With the PAI-1 Expression

As previous report suggested, PAI-1 facilitates PD-L1 endocytosis of melanoma cells to abrogate the efficacy of anti-PD-L1Abs (2), next we evaluated the PD-L1 expression on melanoma cells in each patient in cohort 1. IHC staining for PD-L1 was performed, and the percentage of PD-L1+ cells per all melanoma cells was automatically counted and quantitatively analyzed by BZ-X800. Percentage of PD-L1 expression on melanoma cells was significantly higher in the low PAI-1 expression group (**Figure 1E**) compared to that of the high PAI-1 expression group (**Figure 1F**) (p=0.0168) (**Figure 1**).

### Serum PAI-1 Levels in Melanoma Patients Treated With Anti-PD1 Abs

**Figure 2:**
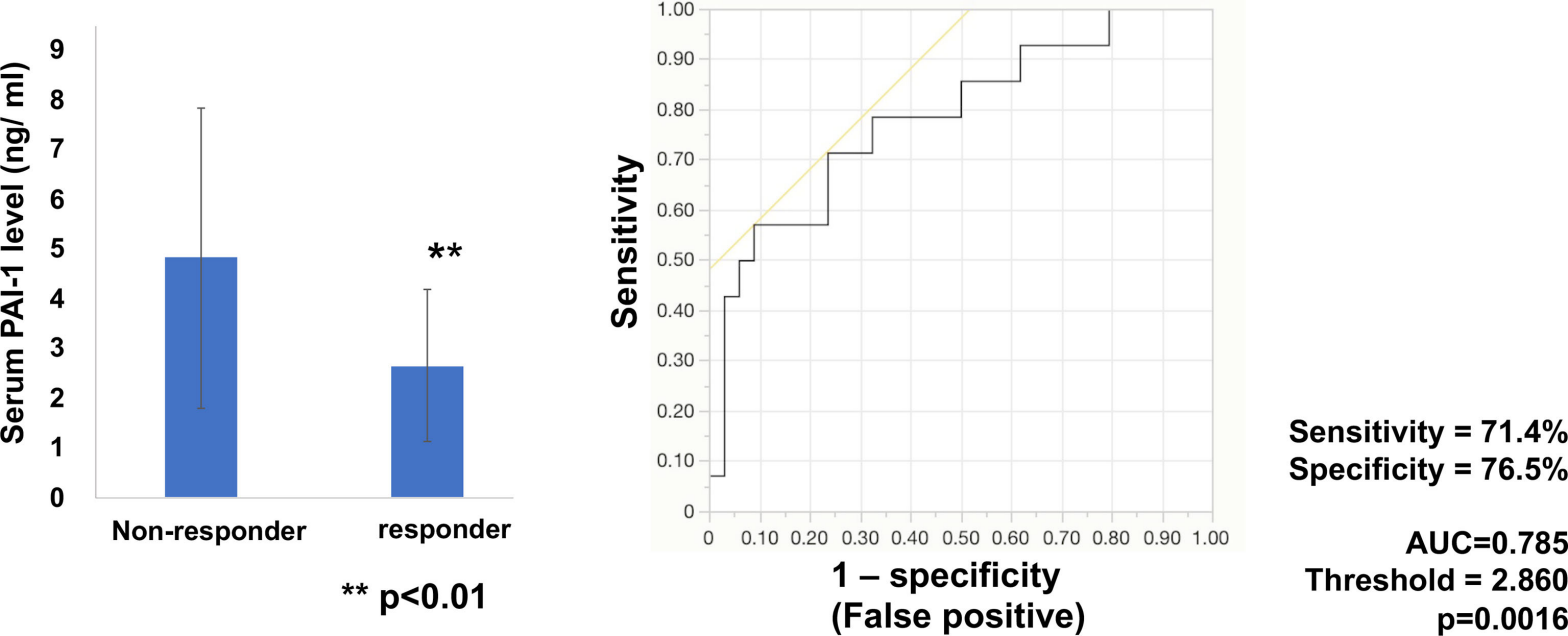
Serum levels of PAI-1 and the ROC curve in melanoma. Mean serum levels of PAI-1 in non-responders (n=34) and responders (n=14) at day 0. The ROC curve was used to calculate cut-offs for PAI-1 serum levels and the AUC. Cut-offs were determined to distinguish responders from non-responders using Youden’s index.

Next, to determine whether baseline serum levels of PAI-1 might be associated with early response in melanoma patients treated with anti-PD1 Abs, PAI-1 levels were evaluated in 49 patients (cohort 2) with advanced melanoma treated with anti-PD1 Abs. The baseline serum levels of PAI-1 and the best response to anti-PD1 Abs in each patient in cohort 2 are presented in **Table 2**. The threshold value of PAI-1 at baseline to distinguish responders from non-responders was 2.860 ng/ml. The sensitivity and specificity of the baseline serum PAI-1 in advanced melanoma were 71.4% and 76.5%, respectively (p=0.0016; **Figure 2**). High baseline serum levels of PAI-1 were significantly correlated with resistance to nivolumab in patients with advanced melanoma (p=0.0020) (**Figure 2**).

### CD163+ Tumor-Associated Macrophages (TAMs) and the Efficacies of Anti-PD1 Abs in Melanoma

Since PAI-1 polarized TAMs toward proinflammatory and M2 phenotypes (6), the correlation between the ratio of TAMs among tumor-infiltrating lymphocytes (TILs) and the efficacies of anti-PD1 Abs were evaluated in melanoma patients. IHC staining for CD163, which is a commonly used marker for M2 macrophages (8), was performed, and the percentage of CD163+ TAMs in melanoma was quantitatively analyzed (**Supplementary Figure 1**). There was no significant correlation between the ratio of CD163+ macrophages among tumour-infiltrating cells and the efficacy of anti-PD1 Abs for melanoma (p=0.4288).

### Immunomodulatory Effects of PAI-1 on CD163+ Macrophages *In Vitro*

**Figure 3:**
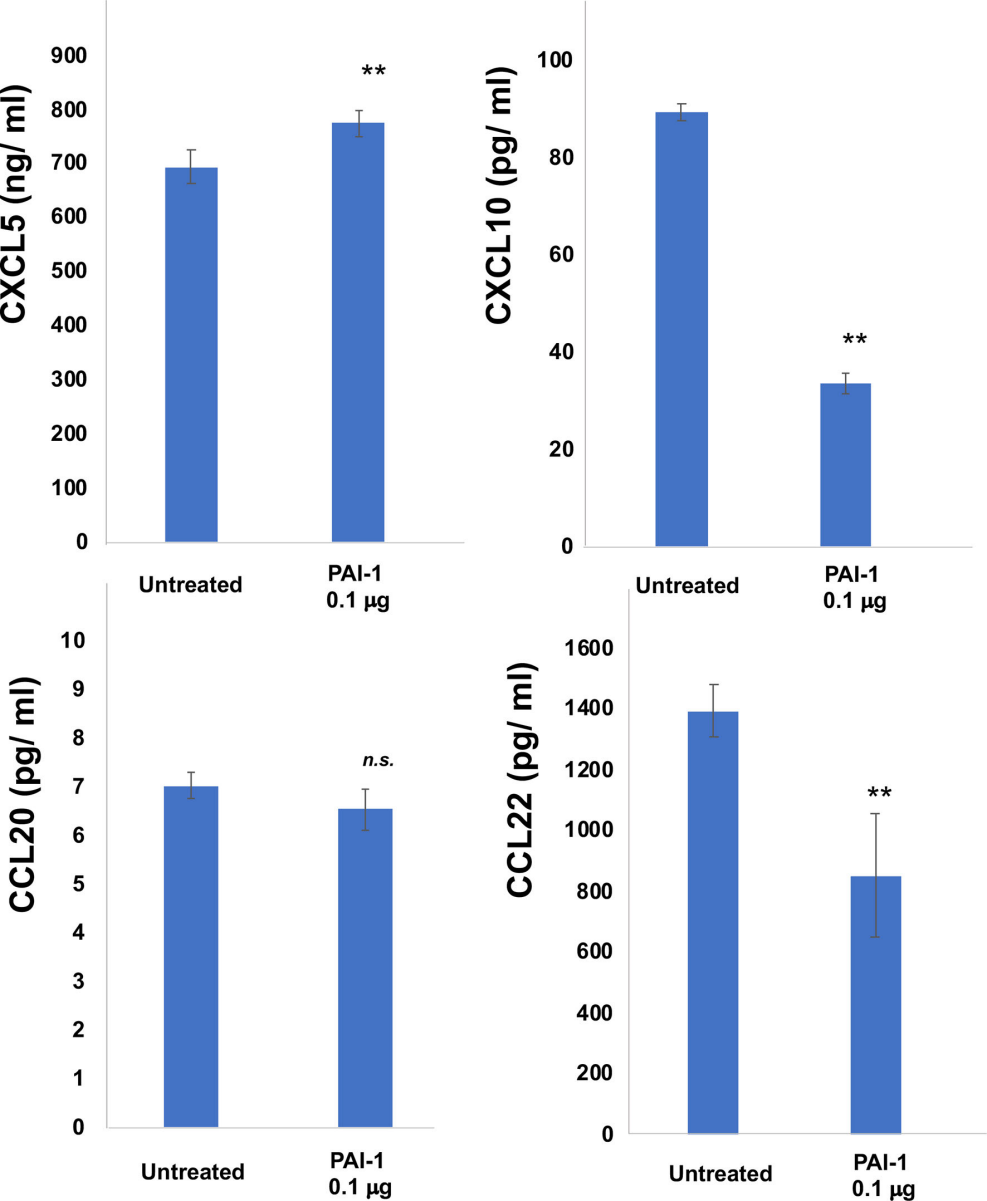
Production of CXCL5, CXCL10, CCL20, and CCL22 by M2 macrophages treated with PAI-1. Culture supernatant from M2 macrophages was harvested as described in Materials and Methods and measured by ELISA (n=3). Data from each donor were obtained from triplicate assays, and mean ± SD values were calculated. Representative data from at least three independent experiments are shown. **p < 0.01, Mann-Whitney U-test; *n.s.*, not significant.

Since the ratio of CD163+ TAMs was not different between the responder group and the non-responder group, to further examine the immunomodulatory roles of PAI-1 on TAMs in the tumor microenvironment, CD163+ M2 macrophages were generated from CD14+ monocytes and stimulated by recombinant PAI-1 *in vitro* (4). PAI-1 decreased the production of CXCL10 and CCL22 (**Figure 3**), suggesting that PAI-1 might decrease the TILs in melanoma. In contrast, PAI-1 increased the production of CXCL5, suggesting that PAI-1 might increase the tumor infiltrating CXCR2+ myeloid-derived suppressor cells (MDSCs) and tumor-associated neutrophils (TANs) (**Figure 3**), both of which are known to be immunosuppressive cells ^12^. There was no significant difference of the production of CCL20, suggesting that PAI-1 might not effect on the production of CCL20 from TAMs (**Figure 3**).

### TILs Were Decreased in PAI-1 Highly Expressing Melanoma

**Figure 4:**
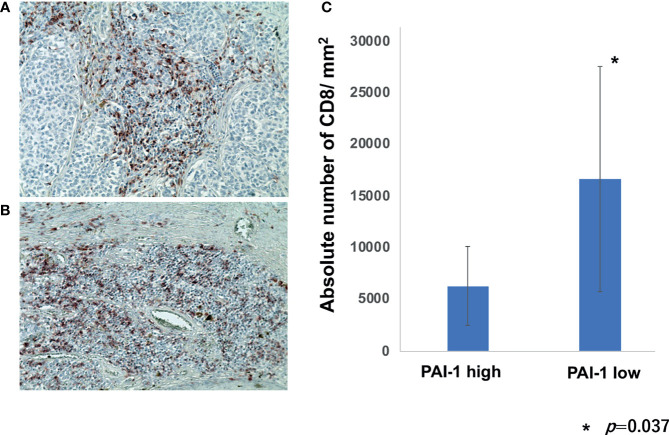
Percentage of CD8+ cells per TILs in melanoma patients. Sections of melanoma from an anti-PD1 Abs in a high PAI-1 expression patient **(A)** and a low PAI-1 expression patient **(B)** were deparaffinized and stained using anti-CD8 antibodies. Sections were developed with liquid permanent red. The percentages of CD8+ cells per TILs were automatically counted by BZ-X800 **(C)**.

To further confirm immunomodulatory effects of PAI-1 on TAMs in melanoma patients, we employed IHC staining for CD8 in each patient in cohort 1. The percentage of CD8+ cells per all tumor-infiltrating leukocytes was automatically counted and quantitatively analyzed by BZ-X800. The absolute number of CD8+ cells per mm^2^ was automatically counted by BZ-X800. The absolute number of CD8+ cells per mm^2^ was significantly increased in a low PAI-1 expression group than that of a high PAI-1 expression group (**Figure 4**).

## Discussion

Plasminogen activator inhibitor-1 (PAI-1) is highly expressed in various types of tumors including melanoma (9), and various pro-tumorigenic functions of PAI-1 in cancer progression and metastasis have been widely reported (9). Among them, tumor-associated inflammation is one of the key pro-tumorigenic functions of PAI-1 (10). Indeed, PAI-1 stimulates the recruitment of fibrosis-inducing cells and macrophages. For example, tumor-derived PAI-1 promotes the migration of monocytes and polarizes tumor-associated macrophages (TAMs) towards proinflammatory phenotypes (7), leading to the production of proinflammatory and angiogenesis-promoting factors such as IL-1, IL-8, CCL2, CCL3, CCL5, and VEGF (11). *Via* an autocrine loop, IL-6 activates signal transduction and activator of transcription (STAT) 3 in monocytes, leading to an increase in the expression of arginase, IL-10, and CD163. Moreover, the expression of these M2-associated markers increases in parallel with immune checkpoints such as B7-homolog superfamily, including B7-H1 (PD-L1) (8). These reports suggested that PAI-1 promotes pro-tumorigenic, immunosuppressive functions through TAMs.

TAMs play various immunosuppressive roles in melanoma (8). For example, TAMs express immune checkpoint modulators that directly suppress activated T cells (12). In addition, TAMs produce various chemokines that attract other immunosuppressive cells such as Tregs and precursors of TAMs to maintain the immunosuppressive tumor microenvironment (8, 13, 14). Notably, TAM-related chemokines, such as CXCL5, CXCL10, and CCL22, as well as a TAM-activation marker, soluble (s)CD163, could be predictive markers for efficacy and immune-related adverse events (irAEs) in anti-PD1 Abs-treated advanced melanoma patients (15–17). In aggregate, TAMs and TAM-related factors could be important biomarkers to predict the efficacies of anti-PD1 Abs in advanced melanoma patients.

From the above findings, we hypothesized that PAI-1 expression on melanoma cells is correlated with the efficacy of anti-PD1 Abs for unresectable melanoma patients. Indeed, the present data suggested that both the expression levels of PAI-1 on melanoma cells and serum PAI-1 levels were significantly correlated with the efficacy of anti-PD1 Abs in advanced melanoma patients. Since the efficacy of anti-PD1 Abs correlates with the number of TILs at the tumor site in various cancers (5), the decreased chemokine production from TAMs might abrogate the anti-tumor immune response against melanoma. In addition, PAI-1 also increased CXCL5 production, suggesting that PAI-1 might increase the immunosuppressive CXCR2+ MDSCs and TANs. Moreover, as a previous report suggested, PAI-1 decreases PD-L1 expression on melanoma cells to abrogate the efficacy of anti-PD-L1 Abs in mouse melanoma models, suggesting another mechanism of inducing tolerance to anti-PD1 Abs (2). Indeed, The PD-L1 expression in a low PAI-1 expression group is significantly higher than that in a high PAI-1 expression group. Furthermore, the number of CD8+ cells in a low PAI-1 expression group was significantly increased than that in a high PAI-1 expression group. In aggregate, PAI-1 induces resistance to anti-PD1 Abs in melanoma, and co-administration of PAI-1 inhibitors might improve the anti-melanoma effects of anti-PD1 Abs. To prove this hypothesis, from September 2021, we have started a phase II study investigating the safety and efficacy of TM5614, a novel PAI-1 inhibitor with better oral bioavailability that selectively inhibits PAI-1 activity (18), in combination with nivolumab in the treatment of unresectable malignant melanoma (jRCT2021210029).

## Data Availability Statement

The original contributions presented in the study are included in the article/**Supplementary Material**. Further inquiries can be directed to the corresponding author.

## Ethics Statement

The protocol was approved by the institutional review board of Tohoku University Hospital. This study was approved by the ethics committee of Tohoku University Graduate School of Medicine, Sendai, Japan (2020-1-759). The patients/participants provided their written informed consent to participate in this study.

## Author Contributions

TH and TF designed the research study. KO and TF collected and analyzed the data. KO, YK, and TF treated the patients and collected the clinical data and samples. TF wrote the manuscript. TF supervised the study. All authors contributed to the article and approved the submitted version.

## Funding

This study was supported in part by the Japan Agency for Medical Research and Development (21ym0126041h0001).

## Conflict of Interest

The authors declare that the research was conducted in the absence of any commercial or financial relationships that could be construed as a potential conflict of interest.

## Publisher’s Note

All claims expressed in this article are solely those of the authors and do not necessarily represent those of their affiliated organizations, or those of the publisher, the editors and the reviewers. Any product that may be evaluated in this article, or claim that may be made by its manufacturer, is not guaranteed or endorsed by the publisher.

## Supplementary Material

Supplementary Figure 1 Sections of melanoma from an anti-PD1 Abs non-responding patient **(A)** and a responding patient **(B)** were deparaffinized and stained using anti-PAI-1 antibodies. Sections were developed with liquid permanent red. The percentages of IHC-positive cells per all tumor-infiltrating cells were automatically counted by BZ-X800 **(C)**. Scale bar, 100 µm.
