# Supplementary figures and images for: Plasminogen Activating Inhibitor-1 Might Predict the Efficacy of Anti-PD1 Antibody in Advanced Melanoma Patients
Source: Front Oncol. 2021 Nov 29;11:798385. doi: 10.3389/fonc.2021.798385 (PMC8666429; doi:10.3389/fonc.2021.798385)

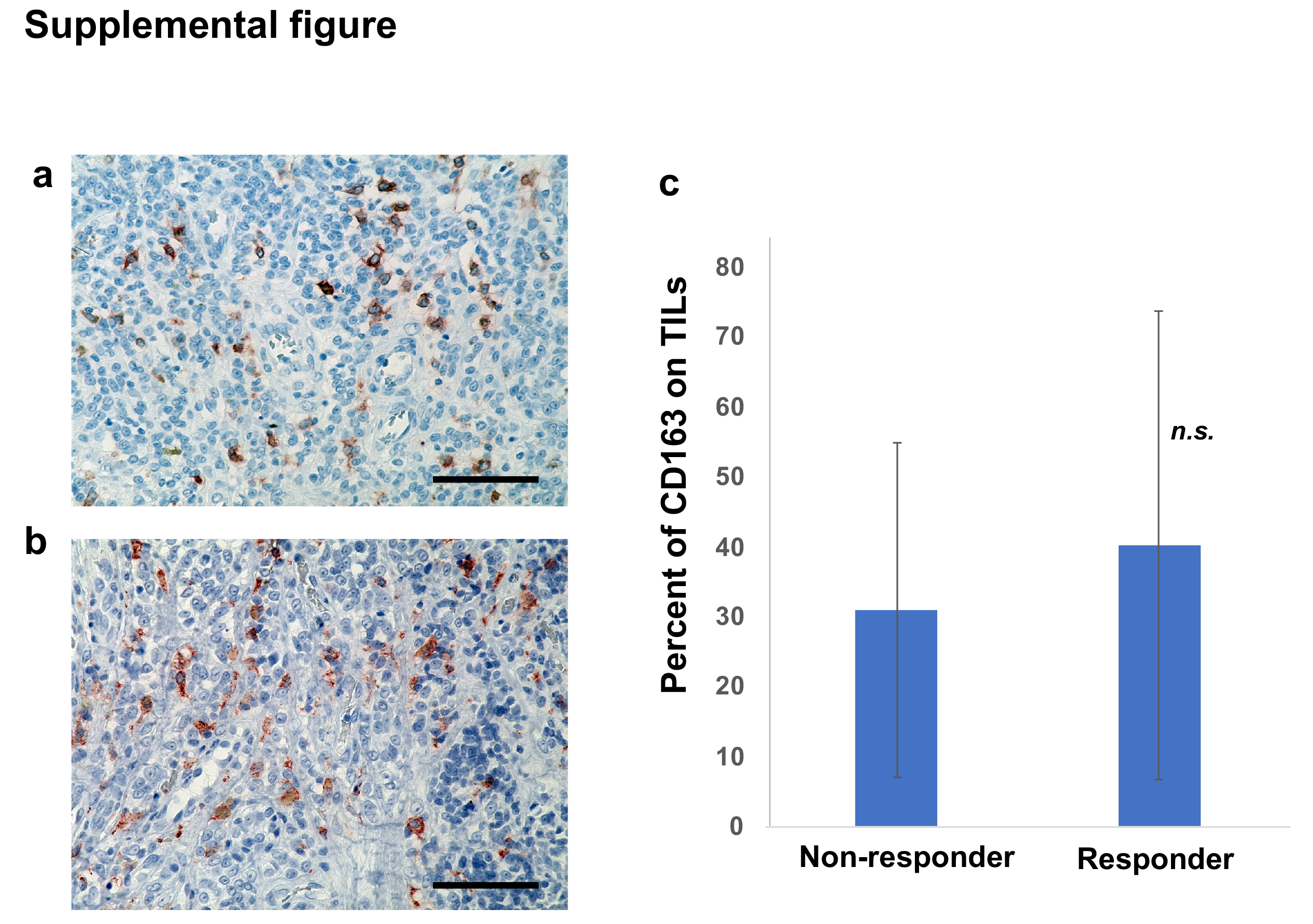

Supplement: Supplementary Figure 1 — Sections of melanoma from an anti-PD1 Abs non-responding patient (A) and a responding patient (B) were deparaffinized and stained using anti-PAI-1 antibodies. Sections were developed with liquid permanent red. The percentages of IHC-positive cells per all tumor-infiltrating cells were automatically counted by BZ-X800 (C). Scale bar, 100 µm. [file Image_1.tif]
